# One-year assessment of in-hospital cardiac arrest

**DOI:** 10.1186/cc13683

**Published:** 2014-03-17

**Authors:** M Ahmed, A Kochhar, O Rose

**Affiliations:** 1 Univeristy Hospital Lewisham, London, UK

## Introduction

This retrospective audit evaluated adult patients who suffered in-hospital cardiac arrest (IHCA) against the recent National Confidential Enquiry into Patient Outcome and Death (NCEPOD) report [1]. It looked specifically at the recognition of the acutely unwell, the interventions made, the decisions taken from admission through to the post-arrest period and the outcomes following cardiopulmonary resuscitation (CPR). The audit aims to guide future improvements in preventing cardiac arrest and enhancing end-of-life care decision-making.

## Methods

Medical notes of adult patients suffering IHCA, over a 1-year period, were identified and data were collected using a validated NCEPOD audit tool. These data included patient demographics, initial clerking and consultant review, patient care during the 48 hours prior to cardiac arrest, the resuscitation status of the patient, the resuscitation attempt, the post-cardiac arrest care and survival to discharge rates.

## Results

Medical notes were available for assessment for 69 out of the 82 patients that were identified as having IHCA between 1 October 2011 and 30 September 2012. The frequency of IHCA showed no correlation to day of the week or month. Initial clerking was incomplete in history- taking (16% vs. 14% in NCEPOD) and in examination (46% vs. 24% in NCEPOD). The majority of patients were appropriately escalated in a timely fashion (94% vs. 82% in NCEPOD), but first consultant review was delayed beyond 12 hours in 49% of cases (48% in NCEPOD). A total 81% of patients suffered cardiac arrest 24 hours after admission (68% in NCEPOD). Warning signs for cardiac arrest were considered present in 59% of cases (75% in NCEPOD), with a significant proportion going unrecognised (27%) despite multiple medical reviews. Out-ofhours CPR attempts (68% vs. 59% in NCEPOD) seemed be associated with poorer survival. The survival to discharge rate after in-hospital cardiac arrest was 10.1%. This compares with 14.6% in the NCEPOD data and 20% in larger studies [2]. Ninety per cent of patients had no documentation of resuscitation status (78% in NCEPOD).

## Conclusion

The results from this audit highlight persistent deficiencies in the care pathway for the acutely unwell patient. Improvement will be focused on earlier consultant review and better recognition of warning signs with appropriate action taken. Furthermore, earlier routine and senior clinician-led discussions on appropriate end-of-life care are vital.
